# Une cardiomyopathie dilatée réversible induite par l’hypocalcémie secondaire à une hypoparathyroïdie post thyroïdectomie totale: à propos d’un cas

**DOI:** 10.11604/pamj.2019.32.34.13886

**Published:** 2019-01-17

**Authors:** Rakotoniaina Masinarivo Daniella, Ramiandrisoa Ritchy, Randriamihangy Avisoa Narindra, Solofonirina Rakotoarimanana, Nirina Rabearivony

**Affiliations:** 1 Service de Cardiologie, CHU Joseph Raseta Befelatanana, Antananarivo, Madagascar; 2 Service des Urgences et Soins Intensifs en Cardiologie, CHU Joseph Raseta Befelatanana, Antananarivo, Madagascar; 3 Service de Cardiologie, CHU Mahavoky Atsimo, Mahajanga, Madagascar

**Keywords:** Hypoparathyroïdie, hypocalcémie, cardiomyopathie dilatée, Hypoparathyroidism, hypocalcaemia, dilated cardiomyopathy

## Abstract

Nous rapportons un cas de cardiomyopathie dilatée d'origine hypocalcémique secondaire à une hypoparathyroïdie, chez une femme de 38 ans. Sa calcémie était effondrée à 30 mg/L et l'échocardiographie avait montré une cardiomyopathie dilatée hypokinétique avec baisse de la fraction d'éjection du ventricule gauche à 31,4%. Elle a reçu une calcithérapie avec de la vitamine D3 et l'évolution était marquée par la normalisation de la taille des cavités cardiaques et de la fraction d'éjection ventriculaire gauche (FEVG) après normocalcémie.

## Abstract (en)

We here report the case of a 38-year old woman with dilated cardiomyopathy induced by hypocalcaemia secondary to hypoparathyroidism. The patient had low calcium level (30 mg/L) and echocardiography showed dilated-hypokinetic cardiomyopathy with reduced left ventricular ejection fraction (31.4%). She received calcitherapy associated with vitamin D3 and her evolution was marked by the normalization of the size of the cardiac cavities and of the left ventricular ejection fraction after normocalcemia.

## Introduction

L'hypoparathyroïdie post-thyroïdectomie totale constitue la première cause d'hypocalcémie chez l'adulte. Cette dernière peut être asymptomatique mais en raison de l'importance du calcium dans le couplage électro-mécanique, elle peut provoquer une dysfonction myocardique qui peut aller jusqu'à une insuffisance cardiaque grave [1].

## Patient et observation

Une jeune femme de 38 ans était admise dans le service de cardiologie, CHU Joseph Raseta Befelatanana Antananarivo pour une dyspnée NYHA IV évoluant depuis 3 semaines avant son admission et des crampes musculaires à répétitions évoluant depuis 6 mois. Elle n'a pas d'antécédent particulier à part une notion de thyroïdectomie totale 3ans auparavant. L'examen physique à l'entrée a montré à 130/80 mmHg, une fréquence cardiaque à 95 bpm et une fréquence respiratoire à 33 cpm avec Sa02 à 92%. La patiente était apyrétique. L'examen cardio-vasculaire a retrouvé un bruit de galop gauche, la présence de turgescence des veines jugulaire. L'auscultation pulmonaire objectivait des râles crépitants aux niveaux des deux bases pulmonaires. Aux bilans biologiques, la patiente avait une hypocalcémie à 30 mg/l, une baisse de la T4 libre à 2,39 pg/ml et une élévation de la TSH à 8,48 μUI/ml. La NFS, la CRP, la glycémie, la créatininémie et l'ionogramme sanguin étaient normaux. L'échocardiographie et doppler ont objectivé une cardiomyopathie dilatée hypokinétique avec FEVG à 31,4% avec pression de remplissage élevée. La patiente avait reçu comme traitement du calcium avec de la vitamine D3 à part la substitution hormonale par Levothyrox et l'évolution était marquée par la normalisation de la taille des cavités cardiaques et de la FEVG après normocalcémie c'est-à-dire après 3 mois de traitement.

## Discussion

L'hypocalcémie est une cause réversible très rare de la CMD pouvant intéresser les deux ventricules [2]. Les symptômes cliniques sont ceux de l'insuffisance cardiaque typiques associés aux signes de l'hypocalcémie. La base du traitement consiste surtout à la correction de l'hypocalcémie par supplémentation en calcium et de la vitamine D [3]. L'évolution est favorable dans la majorité des cas comme celui de notre patiente au bout d'une période très variable selon les cas rapportés dans la littérature, néanmoins, il existe quelque rares cas rapportés où l'évolution reste défavorable [4].

## Conclusion

Le CMD constitue une complication redoutable mais réversible de l'hypoparathyroïdie en rapport avec l'hypocalcémie. Un dépistage systématique basé sur le bilan phospho-calcique doit être effectué après toute chirurgie thyroîdienne. Il faut y penser devant tous patients atteints d'insuffisance cardiaque dans un contexte d'hypocalcémie.

## Conflits d’intérêts

Les auteurs ne déclarent aucun conflit d´intérêts.
